# Haplotype Analysis and Linkage Disequilibrium at Five Loci in *Eragrostis tef*

**DOI:** 10.1534/g3.111.001511

**Published:** 2012-03-01

**Authors:** Shavannor M. Smith, Yinan Yuan, Andrew N. Doust, Jeffrey L. Bennetzen

**Affiliations:** * Department of Genetics, The University of Georgia, Athens, Georgia 30602; † Department of Plant Pathology, The University of Georgia, Athens, Georgia 30602; ‡ Department of Biological Sciences, Purdue University, West Lafayette, Indiana 47907; § Department of Forest Resources and Environmental Science, Michigan Technological University, Houghton, Michigan 49931; ** Department of Botany, Oklahoma State University, Stillwater, Oklahoma 74078

**Keywords:** dwarfing genes, genetic diversity, homeologous loci, millet, tetraploidy

## Abstract

*Eragrostis tef* (Zucc.), a member of the Chloridoideae subfamily of grasses, is one of the most important food crops in Ethiopia. Lodging is the most important production problem in tef. The *rht1* and *sd1* dwarfing genes have been useful for improving lodging resistance in wheat and rice, respectively, in what has been known as the “Green Revolution.” All homologs of *rht1* and *sd1* were cloned and sequenced from 31 tef accessions collected from across Ethiopia. The allotetraploid tef genome was found to carry two *rht1* homologs. From sequence variation between these two putative homologs, an approximate ancestral divergence date of 6.4 million years ago was calculated for the two genomes within tef. Three *sd1* homologs were identified in tef, with unknown orthologous/paralogous relationships. The genetic diversity in the 31 studied accessions was organized into a relatively small number of haplotypes (2−4) for four of these genes, whereas one *rht1* homeologue exhibited 10 haplotypes. A low level of nucleotide diversity was observed at all loci. Linkage disequilibrium analysis demonstrated strong linkage disequilibrium, extending the length of the five genes investigated (2−4 kb), with no significant decline. There was no significant correlation between haplotypes of any of these genes and their recorded site of origin.

*Eragrostis tef* (Zucc.) Trotter, is an allotetraploid (2n = 4x = 40) and one of the most important cereal crops in Ethiopia. Tef belongs to the Chloridoideae subfamily, a lineage of grasses (Poaceae) that has been given very little research attention until recently, with publication of the first linkage maps of tef (Bai *et al.* 1999; Yu *et al.* 2006) and preliminary genetic mapping of finger millet (*Eleusine coracana*) (Gale and Devos 1998; Dida *et al.* 2007). Both the origin and diversity of tef is centered in Ethiopia, and its domestication is predicted to have occurred between 4000 and 1000 BC (Vavilov 1951). Morphological, cytological, molecular, and biochemical assays of several *Eragrostis* species (Chevlier 1940; Porteres 1958; Bekele and Lester 1981, Ingram and Doyle 2003) have provided the consensus opinion that a wild tetraploid, *E. pilosa*, is the direct progenitor of *E. tef*.

The cultivation of tef as a cereal has been primarily in Ethiopia. However, small acreages are harvested in parts of the United States, Australia, and India for livestock feed. The Central Statistical Authority (2010) reported that tef constitutes 19% of the gross grain production of all the major cereal cultivated in the country and 24% of the total acreage of cereals planted in Ethiopia. This broad cultivation can be attributed to the many desirable characteristics this crop possesses, including tolerance to drought stress and water logging, exceptional nutritional value, and relatively few postproduction problems with pests or diseases.

Although tef is grown primarily for human consumption and is featured in approximately two-thirds of the Ethiopian diet, its national average grain yield is relatively low, about 0.95 tons per hectare (Assefa *et al.* 2001). This can mainly be attributed to the low yield potential of unimproved varieties and susceptibility to lodging. Modern research began on tef only in the late 1950s. It was not until 1995 that the first 10 improved varieties of tef were released (Tefera *et al.* 1995). Five varieties were the result of direct selection on the basis of their performance in farmers’ fields, and five were developed through intraspecific hybridization of varieties selected from the tef germplasm collection maintained at the Debre Zeit Agricultural Research Center in Ethiopia. The process of evaluating and characterizing tef germplasm is far from complete, hindering the use of this crop’s genetic resources. For this reason, >80% of Ethiopian farmers use traditional landraces as a seed source for production.

Lodging susceptibility is the most significant production problem for tef. In an average year, 17% of tef grain is lost nationally as the result of lodging, whereas some areas routinely experience losses >50% (Ketema 1993). Lodging in tef is usually attributable to the bending of the thin stalk rather than by breakage of the stalk or uprooting of the plant. This causes the grain to rest on the ground, where it may spoil or be missed during harvest. It is likely that lodging also inhibits crop improvement in the direction of panicles with more or larger seed, as larger panicles cause plants to be more prone to lodging. Moreover, adding more inputs (*e.g.*, fertilizer) to tef is observed to increase plant size, thereby increasing lodging, and leading to a decreased grain yield. These factors together make lodging resistance the single most important improvement that is needed for tef.

During the 1960s, lodging became a serious problem in some major cereal crops such as rice and wheat, when they were grown under high input regimens. The higher grain production was accompanied by longer stems that were unable to support the heavy grain. This problem was circumvented by the introduction of semidwarf varieties into rice and wheat breeding programs, leading to the greatly increased cereal production coined the Green Revolution. Mutations in a small subset of dwarfing genes lead to semidwarf varieties that are less sensitive to lodging and respond to nitrogen fertilizer inputs by increasing the percentage of photosynthate converted into grain rather than vegetative materials, thus increasing grain production. Because of the high yield loss resulting from lodging susceptibility and the manner by which tef lodges, bending of the whole plant structure, tef is a particularly appropriate target for improvement in crop yield with the use of semidwarf varieties.

Although many dwarfing genes have been identified in cereals, most have proven to be useless for crop improvement because of their negative effects on fertility and/or other components of yield. The two best-characterized dwarfing genes that have been proven useful to make productive semidwarf varieties are the *sd1* (semidwarf) and *rht1* (reduced height) genes from rice and wheat, respectively (Ashikari *et al.* 2002; Ellis *et al.* 2002). Hence, *sd1* and *rht1* are suitable candidates to evaluate their contribution to plant height in tef and their potential to transform tef plant architecture into a lodging-resistant plant type. It has been shown that mutations in these dwarfing genes interfere with the gibberellin signaling pathway (Peng *et al.* 1999; Monna *et al.* 2002). Gibberellins (otherwise known as gibberellic acids, or GA) are a class of growth regulators that play a significant role in an array of plant developmental and growth processes, including stem elongation (Hooley 1994). The *sd1* gene encodes a GA-20-oxidase, an enzyme involved in the biosynthesis of GA. The *sd1* mutants used in Green Revolution rice contain point mutations within the coding region of a GA-20-oxidase gene that decrease its activity, and thus are moderately defective in GA production. The *rht1* gene encodes a negative regulator of GA signaling. The mutant forms of this gene deployed in Green Revolution wheat are truncated at their N-termini, thus removing the site at which this negative regulation is manifest.

Haplotype analysis provides an understanding of the range of sequence diversity and of the recombination within that genetic diversity that is distributed within any germplasm. For example, allelic diversity at the *Dwarf8* gene, which is associated with quantitative variation of flowering time and plant height, was analyzed in 92 maize inbreds (Thornsberry *et al.* 2001). The results indicated that a group of sequence polymorphisms within *Dwarf8* were observed to associate with differences in flowering time in maize inbreds, and the distribution of nonsynonymous substitutions suggested that *Dwarf8* has been a target of selection.

Linkage disequilibrium (LD) is a characteristic uncovered by haplotype analysis that describes the degree to which recombination redistributes genetic diversity. LD can be used to measure the association of alleles at different loci with particular traits and thus has the potential to identify a single polymorphism within a gene that is responsible for a difference in phenotype (Flint-Garcia *et al.* 2003). As a result, LD analysis has been used extensively in association analysis studies, especially in humans, in whom large progeny sets and controlled crosses are rare. Rates of recombination and mating patterns (inbreeders *vs.* outcrossers, for instance) strongly affect the level and extent of LD. Therefore, most of the issues considered in population genetics are reflected in LD. Haplotype analysis employing LD has been significant in deciphering the genetic bases of many human diseases, including cystic fibrosis (Kerem *et al.* 1989) and Alzheimer disease (Corder 1994). Recently, the use of LD as a genetic tool has expanded to the plant sciences, most notably in maize (Remington *et al.* 2001; Thornsberry *et al.* 2001; Ching *et al.* 2002), Arabidopsis (Hagenblad and Nordborg 2002; Nordborg *et al.* 2002), and soybean (Zhu *et al.* 2003). LD has proven to be a powerful approach in association analyses to identify alleles responsible for variation in agronomically important traits.

As a first step using molecular tools to investigate genetic diversity in *Eragrostis tef*, this study describes diversity, phylogenetic relationships, and LD for five genes that are predicted to be associated with plant height. Tef homologs of *sd1* and *rht1* orthologs were cloned and sequenced from 31 tef accessions. The results indicate relatively little genetic diversity within tef and that this diversity is distributed in a small number of haplotypes that have experienced very little recombination.

## MATERIALS AND METHODS

### Plant material

A total of 31 *E. tef* germplasm accessions were obtained from the U.S.D.A. National Plant Germplasm System, with many originating from western and central Ethiopia (Table 1). Fourteen accessions were grouped into four sets based on the regions from which the accessions were originally collected. The region of origin was unknown for the other 17 accessions used in these experiments. These 31 accessions were chosen for this study because they exhibit a broad range of morphological variation for several important traits, including lodging, plant height, panicle length, first and second internode length, and culm length (Assefa *et al.* 1999, 2000, 2001). The accessions range from 26 to 54 days for heading, 62 to 117 days to reach maturity, 14 to 65 cm for panicle length, 7 to 42 cm for peduncle length, 11 to 82 cm for culm length, and 31 to 155 cm for plant height.

**Table 1:** Region of origin of *Eragrostis tef* accessions collected from Ethiopia

Accession Number	Cultivar Name	Region of Origin	Accession Number	Cultivar Name	Region of Origin
1. 243909	Sergeyna	Unknown	17. 524434	Addisie	Shewa
2. 358594	Red tef 10	“	18. 524435	Alba	Shewa
3. 358595	22916	“	19. 524436	Balami	Shewa
4. 358596	22917	“	20. 524437	Beten	Shewa
5. 494247	P-44	“	21. 524438	Dabbi	Gojam
6. 494250	P-53	“	22. 524439	Enatite	Shewa
7. 494289	R-5	“	23. 524440	Gea-lamie	Wellega
8. 494298	R-236	“	24. 524441	Gommadie	Keffa
9. 494324	R-342	“	25. 524442	Karadebi	Wellega
10. 494345	450-B	“	26. 524443	Manyi	Shewa
11. 494365	470-B	“	27. 524444	Rosea	Shewa
12. 494366	471-B	“	28. 524445	Tullu Nasy	Wellega
13. 494445	550-B	“	29. 524446	Variegata	Wellega
14. 494487	592-B	“	30. 557456	DZ-01-354	Unknown
15. 494493	598-B	“	31. 557457	Red dabi	“
16. 524433	Ada	Shewa			

Colors designate region of origin and correspond to accessions represented in Neighbor-joining trees (Figures 3−7).

### Candidate gene sequences and degenerate primer design

Sequences of the *rht1* and *sd1* genes in rice, wheat, and other species were aligned to design primers for amplifying their homologs in tef. Tef is a highly self-pollinating crop with natural out-crossing of only ∼0.2% to 1.0%. Therefore, tef accessions are usually homozygous. In addition, several clones (PCR-derived and genomic) were isolated and sequenced at various stages of the gene isolation process to ensure that the tef accessions were homozygous. Several degenerate primers were designed from the most conserved regions among the *rht1* and *sd1* orthologs, based on standard degenerate primer design. Amplification of primer pairs was tested on genomic DNA isolated from an *E. tef* accession (PI 557456). The resulting PCR products were cloned (pCR 4-TOPO; Invitrogen, Carlsbad, CA) and sequenced. Sequences were analyzed by using BLASTN on the nonredundant database at Genbank. PCR amplification products with high identify to *rht1* and *sd1* orthologs were gel purified (QIAGEN, Valencia, CA) and used as probes to screen tef genomic libraries for cross-hybridizing clones.

### Genomic library construction and screening

Two *E. tef* genomic DNA libraries were constructed and screened as described in Maniatis *et al.* (1982). Following a standard CTAB DNA isolation protocol, we isolated genomic DNA from leaf tissue collected from tef accession PI 557456, partially digested it with *Sau*3a (NEB, Beverly, MA), and size fractionated it on a sucrose gradient. DNA fragments ranging from 9 to 23 kb were ligated into a *Lambda* DASH II replacement vector and packaged using a *Lambda* DASH II/*Bam*HI Vector Kit as described by the manufacturer (Stratagene, LaJolla, CA). Separate *sd1* and *rht1* probes amplified with degenerate primers designed from rice and wheat orthologs, respectively, were used to screen each tef genomic library for cross-hybridizing clones. Positive bacteriophages were grown and purified using standard protocols (Maniatis *et al.* 1982).

### Cloning genes of interest

Genomic clones that hybridized to the *sd1* or *rht1* probes were digested with *Not*I to identify clones with a greater than 4-kb insert size, thereby potentially carrying a full-length gene. Clones with a >4-kb insert were then aligned into contigs based on *Not*I restriction digestion, Southern blot analysis of clones with probes amplified with *sd1* and *rht1* degenerate primers, and PCR amplification of clones with the same degenerate primers. Clones within the contig that hybridized to the *sd1* or *rht1* probes and amplified with the *sd1* or *rht1* primers were digested with *Not*I, gel purified (QIAGEN) and subcloned into the pBluescript II KS phagemid vector as described by the manufacturer (Stratagene).

### Sequencing genes of interest and primer design

To sequence the *sd1* and *rht1* genomic subclones, the ends of each gene were first sequenced with T3 and T7 universal primers. Conserved primers were designed from the end sequences, and primer sequence walks provided contiguous sequence of both strands of the genes of interest. Both strands were sequenced to improve the accuracy of the edited consensus sequence. Conserved primers were used to sequence the complete *rht1* and *sd1* tef genes. To amplify the *sd1* and *rht1* genes from 30 tef accessions, gene-specific primers were designed from the 5′ UTR and 3′ end of each gene by aligning the sequences and scrutinizing them for nucleotide substitutions unique to each gene. The resulting PCR product lengths for *sd1* and *rht1* are about 4.6kb and 2.3kb, respectively.

### PCR amplification and sequencing

All alleles of the three *sd1* genes and two *rht1* genes were amplified from 30 tef accessions (Table 1) with gene-specific primers using long-range PCR. The amplified regions included the 5′ UTR, coding region, and introns. PCR amplification of genomic DNA templates was performed using Enhanced DNA Polymerase (Stratagene) with approximately 1 minute of extension time per kb. All other PCR parameters were used according to the manufacturer’s recommendations. The PCR products were gel purified (QIAGEN) and cloned into the Invitrogen pCR 4-TOPO cloning vector. Two clones were selected for each *sd1* or *rht1* homolog from each tef accession and sequenced to identify and correct any errors that might have been introduced by the polymerase reaction or sequencing. T3 and T7 universal primers were used to sequence the ends of each clone. Primers designed from conserved regions among the *sd1* and *rht1* genes were utilized to sequence the complete genes.

### DNA sequence analysis

DNA sequences were checked for accuracy and assembled using Phred (base-calling) and Phrap (assembly) (Ewing and Green 1998; Ewing *et al.* 1998). A Phred quality score of 20 (q > 20), or an accuracy of 99.99%, was used as the minimum value for acceptable sequence data (Ewing and Green 1998). To resolve potential errors introduced by the polymerase enzyme or sequencing, chromatograms were scrutinized for all singleton polymorphisms and edited manually as needed. Multiple alignments of *sd1* and *rht1* sequences were generated with the Molecular Evolutionary Genetics Analysis (MEGA version 4.0, Tamura *et al.* 2007) and Genetics Computer Group, sequence analysis software packages. Phylogenetic tree construction was performed with the neighbor-joining method (Saitou and Nei 1987), with distances represented as the number of nucleotide differences. Confidence in the phylogeny was assessed by using one thousand bootstrap replicates. Sequences obtained from genomic subclones, cloned PCR products and PCR-amplified probes were analyzed using BLASTN to verify cloning or amplification of the correct gene. All sequencing was performed in the Genomics and Bioinformatics Sequencing Facility at the University of Georgia. Sequences have been submitted to GenBank (accession numbers: *Tef-rht1-1* (JN793956-JN793986); *Tef-rht1-2* (JN799273-JN799303); *Tef-sd1-1* (JN799304- JN799334); *Tef-sd1-2* (JN799335- JN799365); *Tef-sd1-3* (JN799366- JN799396).

### Statistical analysis

The DnaSP (DNA Sequence Polymorphism) software package version 4.0 (Rozas and Rozas 1997, 1999) was used for analysis of DNA polymorphism, sequence divergence between samples, and recombination between haplotypes. DnaSP was also used for estimation of nucleotide diversity by calculating the average pairwise difference between sequences, Π (Tajima 1983), and the number of segregating sites in a sample, Θ (Watterson 1975). These parameters have expected values of 4Nμ for an autosomal gene of a diploid organism, where N and μ are the effective population size and the mutation rate per nucleotide site per generation, respectively.

Linkage disequilibrium (LD) was calculated as a function of distance between polymorphic sites using the SITES (Hey and Wakeley 1997) and TASSEL (http://www.maizegenetics.net/index.php?page=bioinformatics/index.html) software packages. To quantify and compare LD measurements, squared correlation coefficient between polymorphic sites (r^2^) and standardized disequilibrium coefficient (D′) statistical parameters (Hedrick 1987; Weir 1996) were used. Both recombination and mutation account for the r^2^ measure, whereas D′ is affected only by recombination, not mutational history (Weiss and Clark 2002; Carlson *et al.* 2004). The statistical significance (*P* < 0.001) of pairwise comparisons between polymorphic loci was determined by the Fisher’s exact test and Bonferroni Correction. The extent of LD, as a result of distance across genes, was measured with a fitted regression line (Hill and Weir 1988).

### Divergence dates

Coding sequences of grass orthologs of the *rht1* and *sd1* genes with respective outgroup sequences were obtained by screening with BLAST the tef *rht1* and *sd1* sequences against the BLASTN nonredundant database and against grass genomes at Phytozome (http://www.phytozome.net/). Recovered sequences were converted into protein alignments and aligned using T-Coffee (in Expresso mode using protein structural information) (Armougom *et al.* 2006) and then manually inspected and adjusted in MacClade 4.5 (Maddison and Maddison 2000). The protein alignments were then used to align the nucleotide sequences in MacClade 4.5.

Modeltest version 3.7 (Posada and Crandall 1998) was used in conjunction with Paup* (Swofford 2001) to determine the best model of nucleotide evolution for each data set, both over all positions and with each codon position considered separately.

Phylogenetic analysis was performed under a maximum likelihood (ML) framework in PhyML (Guindon and Gascuel 2003), as implemented in Geneious version 5.4.4 (Drummond *et al.* 2010), with support for clades estimated by analysis of 1000 bootstrapped data sets. Bayesian analysis of the nucleotide data sets was done using Mr Bayes version 3.1 (Huelsenbeck and Ronquist 2001; Ronquist and Huelsenbeck 2003).

Divergence times were estimated using BEAST version 1.6.1 (Drummond *et al.* 2006; Drummond and Rambaut 2007). The validity of a molecular clock, which assumes that substitution rates are constant across the reconstructed tree, was examined by estimating trees using both a strict and relaxed lognormal clock. Results from these runs were compared using Bayes factors in Tracer version 1.5 (Rambaut and Drummond 2007a). A single calibration point of 55 million years ago (mya) for the major diversification of the grass groups (Wolfe *et al.* 1989; Crepet and Feldman 1991), modeled as a lognormal distribution with offset of 55 million years and log mean of 0 and log standard deviation of 1, was used to estimate divergence dates throughout the tree. Runs were performed for 30 million generations, with trees sampled every 3000 generations. Sampled trees were summarized in TreeAnnotator version 1.6.1 (Rambaut and Drummond 2007b) and visualized in Figtree version 1.3.1 (Rambaut and Drummond 2007c).

## RESULTS

### *rht1* and *sd1* homologs in *Eragrostis tef*

**Figure 1:**
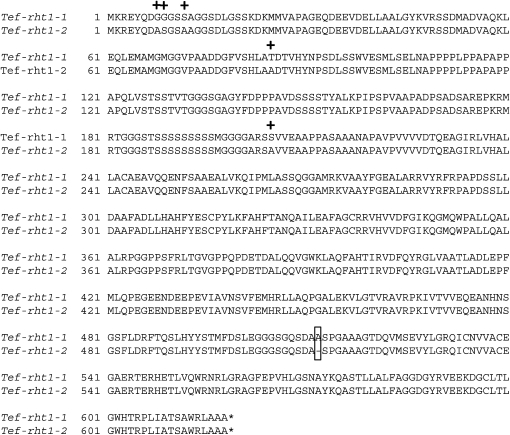
*Tef-rht1* amino acid alignment. Numbers correspond to the amino acid at the beginning of each row with the first amino acid methionine (M) designated as 1. Plus (+) marks indicate amino acid substitutions resulting from nonsynonymous nucleotide changes. Rectangular box indicates an indel. Gene designations: *rht1-1* and *-2* refer to two tef *rht1* homologs. Asterisks (*) correspond to stop codons (TGA).

Twenty recombinant lambda clones from the tef genomic DNA library hybridized to an *rht1* probe amplified with primers designed from a conserved region among cloned *rht1* genes from different species. The clones aligned into two sets based on gel blot hybridization after *Not*I digestion and PCR amplification with *rht1* primers. Sequence analysis of the PCR products confirmed that the clones shared high homology (>90%) with the *rht1* orthologs from other species. Most clones carried individual fragments that amplified with degenerate primers designed throughout the length of the complete *rht1* gene, therefore potentially carrying the full-length gene. A second set of clones yielded an amplification with degenerate primers designed from the conserved 5′ region in *rht1*. Hence, in this second set of clones the 3′ region of the gene is predicted to be truncated by the cloning process. Gel blot hybridization analysis of 31 highly inbred tef accessions always yielded two bands, suggesting the presence of only two *rht1* orthologs in tef (data not shown). Two clones from the full length set were subcloned, completely sequenced, annotated and found to encode two different genes. Because these genes are from a highly inbred tef variety that yields only two gel blot hybridization bands with an *rht1* probe, they are predicted to be orthologous genes (*i.e.*, homeologues) from the two genomes present in the tetraploid tef nucleus. These genes were designated *Tef-rht1-1* and *Tef-rht1-2*. Including the predicted 5′ UTR (~448 bp), coding region (~1.9 kb), and 3′ UTR (~611 bp), the two *rht1* tef genes were observed to be about 2.9 kb in size and to not contain introns in the coding region. The genes share ∼96.4% sequence identity with a total of 117 polymorphic sites. Thirty-six of these polymorphic sites are in the coding region and result in only five amino acid substitutions, all conservative changes (Figure 1). A total of 20 indels ranging from 1 to 8 bp were identified in noncoding regions, whereas only one indel (3bp in size) was identified in the coding region.

**Figure 2:**
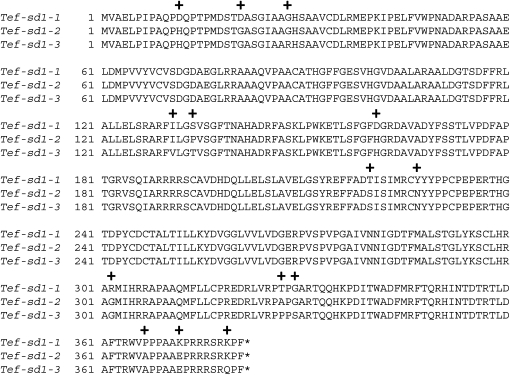
Alignment of predicted protein sequences from *Tef-sd1* homologs. Numbers correspond to the amino acid at the beginning of each row with the first amino acid methionine (M) designated as 1. Plus (+) marks indicate amino acid substitutions resulting from non-synonymous nucleotide changes. Gene designations: *sd1-1*, *-2*, and *-3* refer to three tef *sd1* homologs. Asterisks (*) correspond to stop codons (TGA).

Twenty-eight lambda clones from a second tef genomic library hybridized to an *sd1* probe designed from a conserved region among *sd1* orthologs cloned from different species. Clone inserts were sized by *Not*I digestion, and PCR amplification was performed with primers designed from a conserved region among the rice *sd1* homologs. The resulting 400 bp PCR product was cloned, and 96 clones were sequenced. Based on sequence analysis, the 96 clones grouped into three different classes and showed high homology to the rice *sd1*genes. Therefore, three clones corresponding to the three different classes were selected as putative tef *sd1* homologs, and these were subcloned, sequenced, annotated, and designated *Tef-sd1-1*, *Tef-sd1-2*, and *Tef-sd1-3*. Sequence analysis indicated that the three genes cover ∼4.6 kb each. The 5′ UTR and coding regions are predicted to be about 1.8 kb and 1.1 kb, respectively, for each gene. The coding regions exhibit apparent 98 bp and 1.4 kb introns, located a respective 488 bp and 791 bp downstream of the predicted translation start site. In the ∼4.6-kb region, 70 nucleotide changes were identified among the three genes, 18 of which are located in the coding region. Fourteen amino acid substitutions, all conservative changes, are predicted to result from nucleotide changes in the coding region (Figure 2). Six indels, from 1 to 3 bp, were identified in the noncoding region. Indels were not identified in the *sd1* coding region. *Tef-sd1-2* and *Tef-sd1*-3 shared the greatest sequence similarity (99.2%) with 40 polymorphic sites in a 4.6-kb region. *Tef-sd1-1* and *Tef-sd1-2* are 98.8% identical (53 polymorphic sites). *Tef-sd1-1* and *Tef-sd1-3* share 98.9% sequence identity (49 polymorphic sites). Tef and rice *sd1* homologs were aligned to analyze their sequence homology across the entire gene. Of the three tef *sd1* genes, *Tef-sd1-3* demonstrated the greatest sequence identity (89%) to the rice *sd1* ortholog at the nucleotide level across the 1.1 kb of coding sequence.

### Types, frequency, and distribution of polymorphisms

Sequence data for two tef homeologues (*Tef-rht1-1* and *Tef-rht1-2*) and three *sd1* homologs (*Tef-sd1-1*, *Tef-sd1-2*, and *Tef-sd1-3*) were obtained from 31 tef accessions (Table 1) by PCR amplification using primers specific to each gene. Various nucleotide changes and indels were identified within the population of 31 accessions at each locus in the 5′ UTR and coding region (Table 2). In all cases, only one sequence was identified per accession for each of the five genes, confirming that these lines were all highly inbred.

**Table 2:** Polymorphism and LD data summary

Parameter	*Tef-rht1-1*	*Tef-rht1-2*	*Tef-sd1-1*	*Tef-sd1-2*	*Tef-sd1-3*
Number of tef accessions analyzed	31	31	31	31	31
Number of haplotypes observed	10	4	3	3	2
Total length of region analyzed, bp	2291	2293	4585	4585	4577
Length of coding region analyzed, bp	1857	1854	1065	1065	1065
Length of non-coding region analyzed, bp	434	439	3520	3520	3512
Number of indels	0	3	6	1	9
Number of nucleotide substitutions	32	35	14	17	15
Overall frequency of polymorphic sites per bp	1 per 71.5	1 per 65.5	1 per 327.5	1 per 269.7	1 per 305.1
Frequency of polymorphic sites per bp, coding	1 per 371.4	1 per 123.6	1 per 355	1 per 177.5	1 per 1065
Frequency of polymorphic sites per bp, noncoding	1 per 20.6	1 per 21.9	1 per 320	1 per 320	1 per 250
(π), Average pairwise diversity	0.00382	0.00575	0.00147	0.00182	0.00166
(Θ), Number of segregating sites	0.00350	0.00383	0.00077	0.00093	0.00082
Gene diversity, haplotypes	0.892	0.647	0.671	0.609	0.503
Nonsynonymous amino acid substitutions	4	3	2	3	1
LD pairwise comparisons	465	595	91	136	105
Significant pairwise comparisons*^a^*	112	409	91	136	105
Bonferroni correction*^b^*	17	174	78	136	105
Magnitude of LD*^c^*	0.216	0.472	0.828	0.816	1

LD, linkage disequilibrium

a Fisher’s exact test at *P* < 0.001.

b Correction at *P* < 0.001.

c Measured as a function of distance across loci ranging for 0.0 (no LD) to 1 (complete LD).

Within the 2.3-kb region that was sequenced and compared for the *Tef-rht1-1* and *Tef-rht1-2* loci, a respective 32 and 35 single nucleotide substitutions were identified from these 31 accessions. Compared with the protein-encoding regions, the frequency of nucleotide substitutions was found to be almost 18 times greater in the noncoding regions of *Tef-rht1-1* genes and about six times greater in the noncoding regions of *Tef-rht1-2*. The 15 *Tef-rht1-2* nucleotide substitutions within the coding region contribute only three amino acid replacements, whereas the 23 nucleotide substitutions within the *Tef-rht1-1* gene provide only four predicted amino acid changes. None of these changes is predicted to have a dramatic effect upon protein structure or function.

Single nucleotide substitutions across the 31 accessions were found to range from 20 to 24 in the 4.6-kb segment analyzed in the three tef *sd1* homologs. As with the *rht1* homologs, most of these nucleotide substitutions (>57%) were found in the noncoding region. Also, as in the *rht1* homologs, the majority of the nucleotide substitutions observed in the protein-encoding portions of these genes are predicted to be silent at each locus analyzed. The fewest amino acid changes were observed at the *Tef-sd1-3* locus, with only one nucleotide substitution resulting in an amino acid change.

Indels were only identified in the noncoding region of each locus and rangee from 0 at *Tef-rht1-1* to 9 at *Tef-sd1-3*. Three indels were identified in the 439-bp noncoding region of *Tef-rht1-2*. Conversely, only one indel was identified in a 3.5-kb noncoding region of *Tef-sd1-2*. Single nucleotide indels were found to be the most frequent size class at these five loci.

### Haplotype analysis

**Figure 3:**
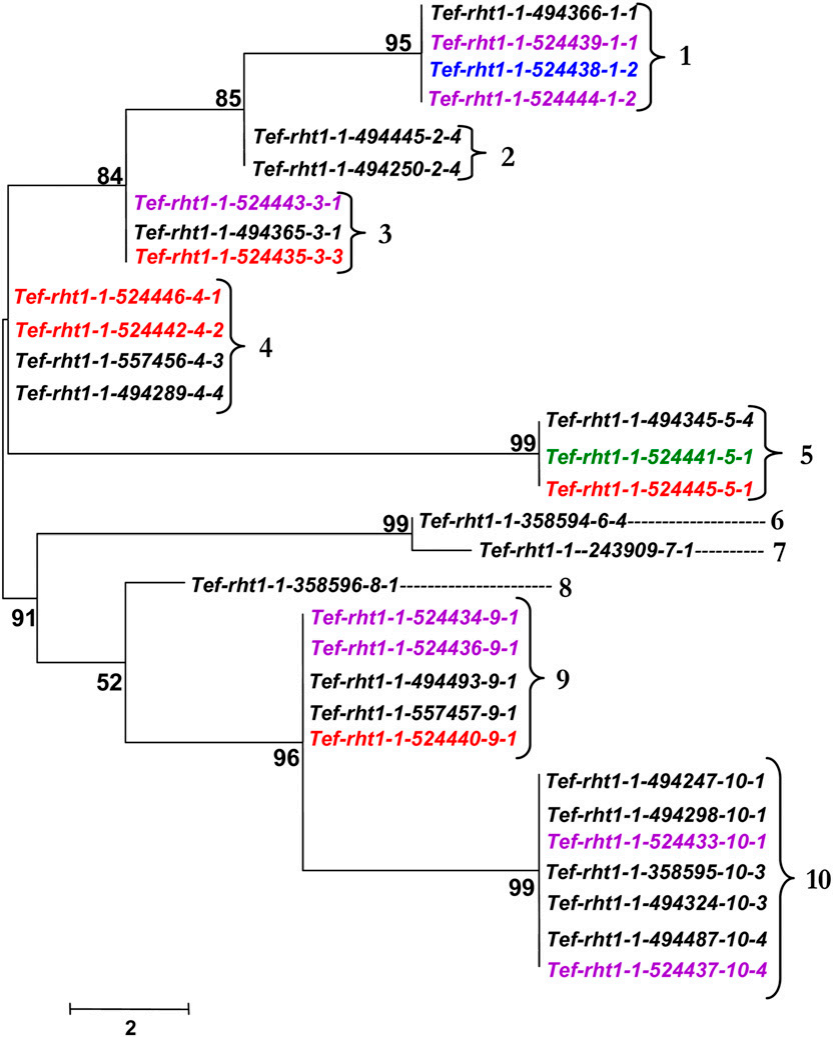
Neighbor-joining tree demonstrating sequence relationships for *Tef-rht1-1* haplotypes. Colors indicate the accession’s region of origin as provided in Table 1. Numbers at nodes indicate the level of branch support (%) with 1000 bootstrap replicates. Numbers at brackets indicate the haplotype designations.

**Figure 4:**
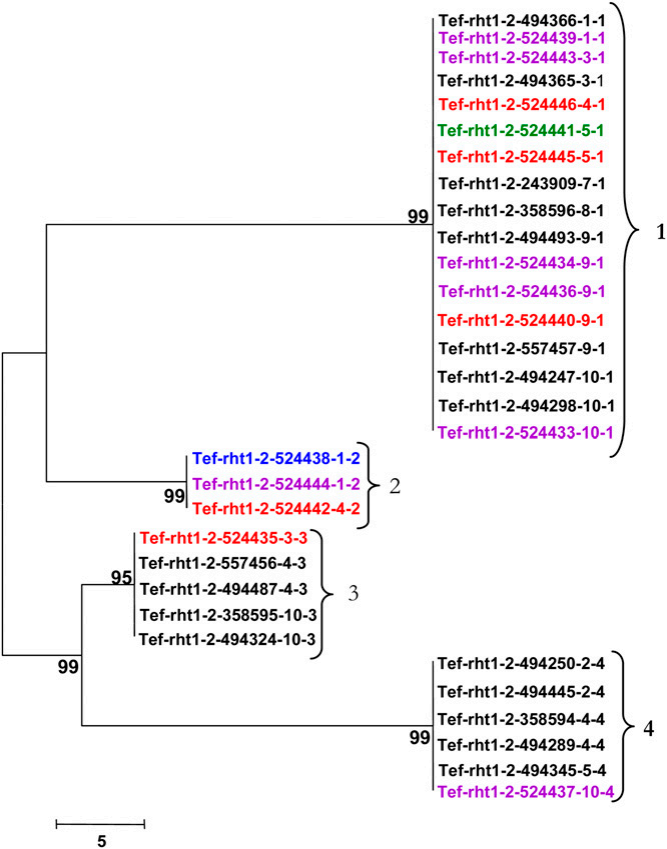
Neighbor-joining tree demonstrating sequence relationships for *Tef-rht1-2*. haplotypes. Colors indicate the accession’s region of origin as indicated in Table 1. Numbers at nodes indicate the level of branch support (%) with 1000 bootstrap replicates. Numbers at brackets indicate haplotype designations.

**Figure 5:**
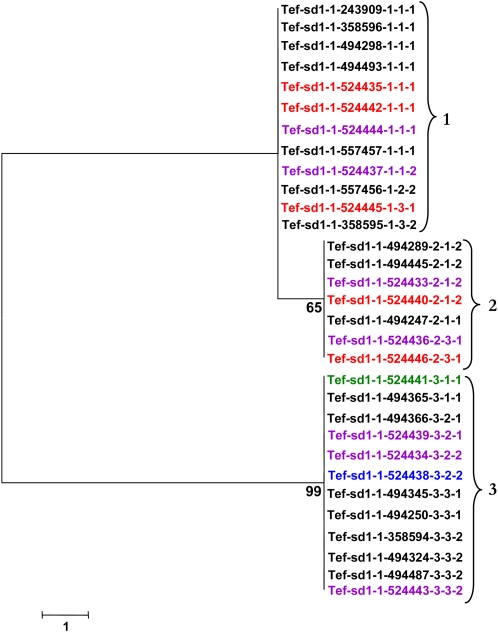
Neighbor-joining tree demonstrating sequence relationships for *Tef-sd1-1* haplotypes. Colors indicate the accession’s region of origin as indicated in Table 1. Numbers at nodes indicate the level of branch support (%) with 1000 bootstrap replicates. Numbers at brackets indicate haplotype designations.

**Figure 6:**
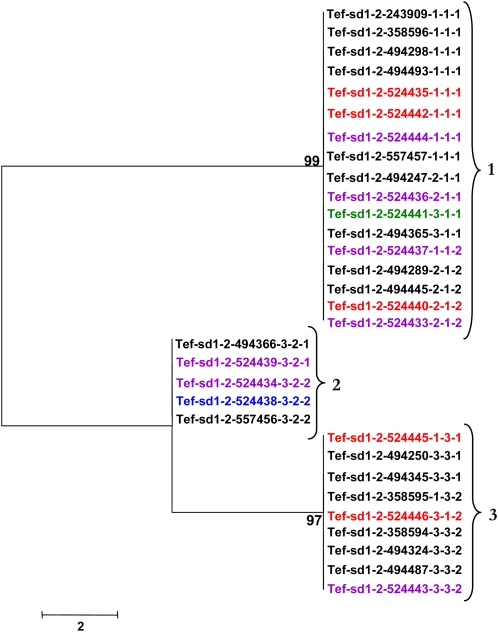
Neighbor-joining tree demonstrating sequence relationships for *Tef-sd1-2* haplotypes. Colors indicate the accession’s region of origin as indicated in Table 1. Numbers at nodes indicate the level of branch support (%) with 1000 bootstrap replicates. Numbers at brackets indicate haplotype designations.

**Figure 7:**
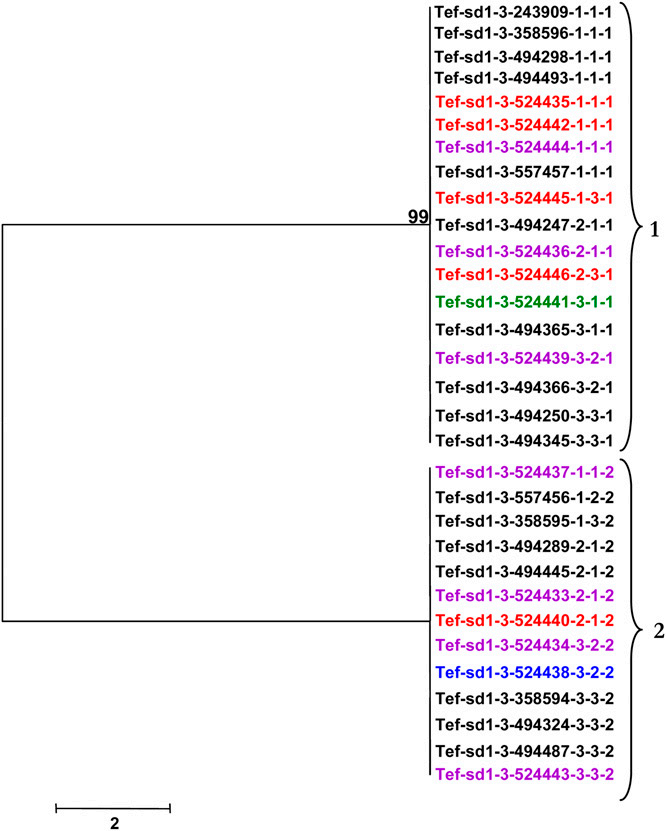
Neighbor-joining tree demonstrating sequence relationships for *Tef-sd1-3* haplotypes. Colors indicate the accession’s region of origin as indicated in Table 1. Numbers at nodes indicate the level of branch support (%) with one-thousand bootstrap replicates. Numbers at brackets indicate haplotype designations.

Neighbor-joining tree construction for *rht1* and *sd1* alleles from each tef accession facilitated the analysis of sequence diversity. This analysis showed the distribution of tef accessions into haplotypes based on sequence differences. The tef accessions are arranged into a relatively small number of haplotypes, ranging from ten haplotypes for *Tef-rht1-1* to two haplotypes for *Tef-sd1-3* (Figures 3−7).

The most distant *Tef-rht1-1* haplotypes are 1 and 10 (Figure 3). These haplotypes are separated by 16 polymorphic sites in a 2.3-kb region and are 99.3% identical. Haplotypes 6 and 7 differ by a single nucleotide and share 99.95% identity. For *Tef-rht1-2* (Figure 4), haplotypes 1 and 4 are 98.83% identical and are the most distant (27 polymorphic sites in a 2.3-kb region). Conversely, haplotypes 3 and 4 share 99.4% identity and differ by 15 nucleotides.

Low nucleotide diversity also is observed for the *sd1* homologs. Haplotypes 1 and 3 are the most distant for *Tef-sd1-1* (Figure 5) with 13 nucleotide differences (99.7% identity) in a 4.6-kb region. Haplotypes 1 and 2 are the most similar, separated by only one nucleotide change. The most divergent haplotypes (1 and 3) for *Tef-sd1-2* carry 17 nucleotide differences (99.7% identity) and the most similar haplotypes are 99.9% identical, with four polymorphic sites (Figure 6). Tef accessions at the *Tef-sd1-2* locus separated into only two distinct haplotypes, differing by 15 nucleotide changes, and are 99.7% identical (Figure 7).

Most haplotypes were found in at least two accessions for each of the five loci. However, the most diverse locus, *Tef-rht1-1*, exhibited three haplotypes (6, 7, and 8) that were only found in one accession each (Figure 3).

There were no monophyletic clades formed with tef accessions all originating from the same Ethiopian region (Figures 3−7). This finding suggests that the distribution of tef accessions into haplotypes is not affected dramatically by the region of origin. However, record-keeping may not have been accurate for these accessions and the number of accessions with annotated geographic origin is small, so a larger and more clearly representative collection of tef accessions should be analyzed to investigate this point. Analysis of a larger number of loci would be needed to detect subtle biases in haplotype distribution.

The possible presence of haplotypes that were found together consistently for *rht1* and *sd1* was analyzed. These haplotypes are defined here as a group (clade) of accessions maintained together for both tef homeologues (*Tef-rht1-1* and *Tef-rht1-2*) or for all three *sd1* (*Tef-sd1-1*, *Tef-sd1-2*, and *Tef-sd1-3*) homologs. Very few consistent associations were found. In the only detected cases, accessions with haplotypes 2 and 9 for *Tef-rht1-1* (Figure 3) were found only with haplotypes 4 and 1, respectively, for *Tef-rht1-2* (Figure 4).

### Nucleotide diversity

The average nucleotide diversity (Θ) for the 5 loci ranges from 0.00147 for *Tef-sd1-1* to 0.00575 for *Tef-rht1-2* (Table 2). *Tef-sd1-1* carries the fewest (0.00077) segregating sites (π) among the three *sd1* genes and *Tef-sd1-2* maintains the most (0.00093). For the two *rht1* homologs, *Tef-rht1-1* carries the fewest segregating (0.00350) sites (π) while *Tef-rht1-2* maintains the most (0.00383). The diversity is greater in the non-coding region for all loci analyzed, except *Tef-sd1-2* where polymorphic sites occur once per 177.5bp (6 sites) in the coding region (1065 bp) and once per 320 bp (11sites) in the non-coding region (3520 bp).

The genetic diversity between haplotypes is quite low, as calculated from the average number of nucleotide differences separating each haplotype (Table 2). The two haplotypes identified for *Tef-sd1-3* demonstrate the lowest (0.503) level of gene diversity among the five loci analyzed, whereas the 10 haplotypes for *Tef-rht1-1* demonstrate the greatest (0.892) level of gene diversity.

### Phylogenetic relationships

Ten sequences of *rht1* orthologs from other grass species were combined with the two tef sequences and with an outgroup sequence from *Arabidopsis*. Fifteen sequences of *sd1* orthologs from other grass species were combined with representatives of the three tef haplotypes and with two *GA20ox1* outgroup sequences from *Zea* and *Sorghum* (Table S1). Alignment of the *rht1* data set was unproblematic, but alignment of the *sd1* data set showed that the three tef haplotypes contained short regions of sequence that were markedly divergent from the conserved positions in the other sequences. However, ML analyses of alignments with divergent regions either included or excluded did not change the detected patterns in relationships, therefore these regions were included in the final analyses. The substitution model for both genes when all positions were analyzed together was the general time reversible model with a gamma correction for rate heterogeneity. Third positions for both genes had somewhat simpler models with either transition (*rht1*) or transversion (*sd1*) rates held equal.

Bayesian and ML analyses of the *rht1* nucleotide data set gave very similar results, with high support for all clades except at the base of the tree (Figure S1). In the Bayesian analysis, the three major grass clades, ehrhartoid, pooid, and panicoid + chloridoid, form a polytomy, whereas in the ML tree there is a moderately supported sister relationship between the pooid and panicoid + chloridoid clades (82% bootstrap value).

Bayesian and ML analyses of the *sd1* nucleotide dataset showed a different pattern to that found in the *rht1* data set. Both analysis methods show the three tef sequences as sister to *Oryza* (Figure S2). The ML analysis shows the tef and *Oryza* clade (clade 1) as sister to a set of sequences from *Brachypodium*, *Setaria*, *Sorghum*, and *Zea* (clade 2). The relationships within clade 2 reflect the tribal relationships shown by the *rht1* data set, with *Brachypodium* (pooid) sister to the panicoids, and, within the panicoids, *Setaria* (tribe Paniceae) sister to *Sorghum* and *Zea* (tribe Andropogoneae). A further clade of *Setaria*, *Sorghum*, *Hordeum* and *Brachypodium* sequences (clade 3) are sister to clade 1 and 2.

The Bayesian analysis of the *sd1* nucleotide data gave similar results to the ML analysis, when the same nucleotide substitution model was used for all three codon positions. However, when the third codon position was allowed to vary independently from the first two positions, clade 2 is no longer found as sister to the tef-*Oryza* clade (clade 1) but is now a paraphyletic assemblage at the base of the tree, with clade 3 sister to the tef-*Oryza* clade. This indicates that the model of sequence evolution used in the analysis has a major effect on the relationships between the groups of species. However, in all cases the tef sequences are strongly supported as sister to the rice *sd1* sequence, but the branch leading from *Oryza* to the three tef sequences is long, reflecting the divergent regions of sequence in the tef *sd1* copies.

### Divergence dates

Dates for divergence of the different clades were calculated only for the *rht1* data set, where it was possible to unequivocally identify orthologous gene copies. Bayes factor tests of a molecular clock analysis indicate that a relaxed lognormal clock fits the data significantly better than a strict clock model. Therefore, a relaxed lognormal clock was used to calculate divergence times for the major grass clades and subclades (Table 3). A similar analysis was performed without sequence data, to make sure that the choice of priors did not bias the phylogenetic analysis. From sequence variation between the two putative *rht1* homologs, an approximate ancestral divergence date of 6.4 mya was calculated for the two genomes within tef (Table 3).

**Table 3:** Divergence times for grass clades on the basis of the *rht1* data set

Species Comparison	Subfamily/Tribe Comparison	Mean divergence time in millions of years*^a^* (Upper and Lower 95% Confidence Interval)
*Zea mays* – *Triticum aestivum*	*Panicoid* – *Pooid*	57.36 (37.32–5.92)
*Zea mays* – *Eragrostis tef*	*Panicoid* – *Chloridoid*	36.47 (20.64–50.54)
*Zea mays* – *Setaria italica*	*Panicoid*: *Andropogoneae* – *Paniceae*	26.86 (13.70–38.71)
*Zea mays* – *Sorghum bicolor*	*Panicoid*: *Andropogoneae*	14.20 (6.57–22.04)
*Zea mays* – *Tripsacum dactyloides*	*Panicoid*: *Andropogoneae*	7.20 (1.92–12.56)
*Sorghum bicolor* – *Saccharum officinalis*	*Panicoid*: *Andropogoneae*	6.00 (1.63–10.23)
*Setaria italica* – *Pennisetum glaucum*	*Panicoid*: *Paniceae*	7.90 (2.02–13.61)
*Triticum aestivum Brachypodium distachyon*	*Pooid*	40.72 (18.85–63.03)
*Triticum aestivum* – *Hordeum vulgare*	*Pooid*	18.19 (4.44–28.96)
*Eragrostis tef 1* – *Eragrostis tef 2*	*Chloridoid*	6.38 (1.51–11.77)

a Dates are based on a fossil divergence date of 55 million years for the divergence between *Zea* and *Oryza*.

### Linkage disequilibrium

**Figure 8:**
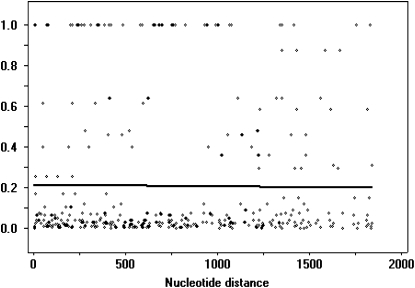
Linkage disequilibrium (LD) scatter plot for *Tef-rht1-2*. LD was measured as a function of distance between polymorphic sites based upon the r^2^ statistical measurement (Y-axis). A total of 595 pairwise comparisons were plotted. Significant comparisons were determined by the Fisher’s exact test (*P* < 0.001) and Bonferroni correction (gray spots). X-axis measurements indicate the nucleotide distance across the gene of interest, with the number 1 corresponding to the first nucleotide at the 5′ end. The logarithmic trend line, indicating the magnitude and extent of LD, was fit to data by the DnaSP program.

LD was measured for all five loci. The r^2^ and D’ LD measure was calculated based on single nucleotide substitutions identified for each locus among 31 tef accessions (Table 2). The r^2^ and D’ LD measurements generated similar results at all loci analyzed; therefore, only graphs for r^2^ are presented. A small number of haplotypes were maintained at each locus, which is characteristic of loci that are in LD. All five loci demonstrate LD extending throughout the full 2.3-kb and 4.5-kb regions analyzed for tef *sd1* and *rth1*, respectively, with no significant decline. The *Tef-rht1-1* locus, which maintains the greatest number of haplotypes among the five loci, shows the lowest magnitude of LD at 0.216 (Figure 8), whereas *Tef-sd1-3*, with only two haplotypes, demonstrates an LD level of 1, denoting complete LD. Recent recombination between haplotypes that could create chimeric haplotypes was not detected at any of the loci analyzed.

## DISCUSSION

### Copy numbers and structures of *rht1* and *sd1* homologs in tef

Thirty-one tef accessions, selected because of their geographic distribution and wide range of morphological and agronomic traits (Assefa *et al.* 1999, 2000, 2001), were analyzed to identify and characterize *rht1* and *sd1* homologs. Tef *rht1* and *sd1* genes were cloned and sequenced from all accessions, and it was observed that their structures (size, intron number and position) were similar to wheat *rht1* (Ellis *et al.* 2002) and rice *sd1* (Ashikari *et al.* 2002), respectively. Tef carries two copies of *rht1* homologs, likely representing separate copies of this locus in the allotetraploid tef genome. In support of these findings, two bands corresponding to each of the tef *rht1* genes were always observed in gel blot hybridization when the 31 highly inbred tef accessions were probed with a 600 bp fragment containing protein-encoding exons from the tef *rht1* gene(s). Wheat also carries only one copy of the gene per genome in tetraploid and hexaploid wheats. Specific dwarfing alleles are located on chromosome 1B (*Rht-B1b*) and chromosome 1D (*Rht-D1b*), and chromosome 1A also has a copy of the gene with no known dwarfing alleles (Ellis *et al.* 2002). This single copy nature is also true for *rht1* orthologs identified in diploid species such as maize (*d8*), rice (*slr1-d*), barley (*sln1*), and Arabidopsis (*GAI*) (Silverstone and Sun 2000; Ikeda *et al.* 2001; Hedden 2003; Hussain and Peng 2003). These data suggest that *rht1* is not often represented as a paralogous gene family, leading to the conclusions that the two *rht1* homologs in tef are probably homeologous copies. If the two tef *rht1* homologs are actually homeologues, as all of these results predict, then the divergence time of approximately 6.4 mya is a prediction of the divergence date of the two ancestral genomes that came together to form the *Eragrostis pilosa* tetraploid. Such a time appears reasonable since the constituent genomes do not appear to be very closely related, based on *rps16* chloroplast and *waxy* nuclear gene trees (Ingram and Doyle 2003).

A small gene family of four gibberellin-20-oxidase (*GA20ox1* to *4*) genes have been described in rice (Sakamoto *et al.* 2004), and GA20 oxidase gene families are also found in sorghum, maize, and barley, with a respective 4, 4, and 3 members (Ogawa *et al.* 2000; Spielmeyer *et al.* 2002; Jia *et al.* 2009). Mutations in one of the four *GA20ox* genes (*GA20ox2*) lead to the semidwarf phenotype in rice. The other three *GA20ox* genes have partial functional redundancy, so the loss-of-function *sd1* mutants are only moderately GA deficient (Hedden 2003). The three *sd1* genes identified in tef are all most closely related to *GA20ox2*, rather than to the other *GA20* oxidases, and all have very similar open reading frames, suggesting that they are functional. Alignment of the three tef *sd1* homologs with the four rice *sd1* genes indicated that *Tef sd1-3* shares the highest sequence identity to the *GA20ox2* semidwarfing gene, but all were at least 79% identical at the nucleotide sequence level within the protein-encoding exons. Additionally, after sequencing and analyzing over 180 *sd1* sequences, we have as yet not isolated other *GA20* oxidases from tef. It is possible that all of the *sd1* homologs of tef examined are paralogues and that one of the homeologous/orthologous copies of the *sd1 Ga20ox* gene was deleted from the tef genome after tetraploidy or in one of the diploid ancestors of the initial tetraploid. It will be necessary to obtain a high-resolution physical map of the tef genome in order to establish whether the tef *sd1* genes are in syntenous positions with those from other species.

### Nucleotide diversity

Our estimates of the levels of nucleotide diversity at these five loci in tef was much lower than that uncovered in comparable studies on a wide variety of genes in many plant species, including maize (Tenaillon *et al.* 2001), *Arabidopsis* (Olsen *et al.* 2002), and sorghum (Li *et al.* 2010). However, the tef gene diversity was greater than that observed for most genes in soybean (Scallon *et al.* 1987; Zhu *et al.* 1995, 2003). The limited genetic diversity observed at the five loci analyzed in tef suggests a narrow genetic base. Several factors may have contributed to a lack of diversity in tef germplasm, including a bottleneck in the wild ancestor population, a small number of plants used to found the domesticated species, and/or a narrowing of acceptable germplasm by human selection. Because a wild tetraploid grass native to Ethiopia, *E. pilosa*, is believed to be the progenitor of tef (Ingram and Doyle 2003), it would be interesting to investigate the genetic diversity in this species and how it has been sampled within the domesticated crop.

The majority of the nucleotide substitutions (point mutations) for the five loci in tef were observed in the noncoding region. Most of the nucleotide changes that occurred in the coding region did not result in amino acid changes, suggesting that these genes are likely to be functional. Moreover, of the twenty indels identified in the five loci, only one (3 bp in size) occurred in the coding region, and this one maintains the coding frame. The indel sizes in the noncoding region were variable, with single nucleotide indels being the most frequent size class, a common observation across all plant species that have been examined. These data are similar to what was described for the functional Green Revolution genes in wheat (*rht1*) and rice (*sd1*) (Peng *et al.* 1999; Ashikari *et al.* 2002; Hedden 2003) and further support the concept that these genes are the functional *rht1* and *sd1* orthologs in tef.

### Haplotypic diversity

A relatively small number of haplotypes were observed for the five loci analyzed in tef. In maize, the various Green Revolution genes exhibit much greater variability. For instance, depending on the gene analyzed and the number of inbreds studied, investigators observed anywhere from a low of eight haplotypes (in an analysis of 71 inbreds) (Andersen *et al.* 2005) to 41 haplotypes (in an analysis of 91 inbreds) (Thornsberry *et al.* 2001). With the broad diversity in origin, agronomic properties and morphologies that were sampled in this study, it is likely that the majority of the most frequent haplotypes for these five genes now have been discovered in tef. There were no cases, for instance, in which the primers specific to each gene were unable to amplify an appropriate allele in each of the 31 accessions; therefore, it is not particularly likely that highly novel haplotypes were missed.

One gene, *Tef-rht1-1*, exhibited 10 haplotypes, more than twice the number of the second most diverse haplotypes (*Tef-rht1-2*, with four haplotypes). Three of these *Tef-rht1-1* haplotypes were found in only one of the 31 studied accessions, suggesting that several more haplotypes are yet to be discovered for this genomic region. It will be interesting to score tef accessions for a great number of traits, to see whether any correlate with any of the *rht1* or *sd1* haplotypes. *Tef-rht1-1* may be tightly linked to some gene that has major effects on differences in local adaptation, like a gene providing race-specific resistance to a microbial or insect pest. A correlation between *rht1* or *sd1* haplotypes and any trait might be caused by the Green Revolution gene itself, but the large LD in tef indicates that many other linked genes would also be candidates for the causal agents.

The 31 tef accessions were not distributed into haplotypes on the bassis of their recorded regions of collection. These results suggest that the ancient and/or current seed trade has accomplished a very broad dissemination of germplasm. Alternatively, or in addition, inaccuracies in the documentation of collection sites for some of these accessions may have obscured region-specific biases in allele frequency. The absence of heterozygotes in any of the tested accessions provides confirmation that extensive gene flow is not likely to occur by pollen dispersal in this inbreeding species.

### LD and recombination

The number of haplotypes at each locus was reflected in the magnitude of LD (r^2^). The *Tef-sd1-3* locus demonstrated LD with only two haplotypes and, as the number of haplotypes increased for the remaining loci, LD decreased. This is similar to what has been reported for maize (Ching *et al.* 2002), Arabidopsis (Nordborg *et al.* 2002), and humans (Daly *et al.* 2001).

Demographic history is an important factor in determining the magnitude of LD among populations of the same species that share similar rates of recombination and mutation. Typically, populations that have expanded rapidly or have remained large for an extended period of time demonstrate a low magnitude of LD, whereas small populations or those that have experienced recent population bottlenecks demonstrate higher levels of LD (Gaut and Long 2003; Mueller 2004). The high LD observed at all tef loci predicts a history of small and isolated populations, which seems unlikely for the current crop but may have been the status of the undomesticated ancestor. None of the tef accessions was distributed into haplotypes on the basis of their recorded regions of collection, suggesting that opportunities for the formation of hybrids should be high. A low number of haplotypes for most of the genes suggests a severe bottleneck at some time prior to or during the development of the domesticated species. In stark contrast, the numerous haplotypes at *rht1-1* suggest that there was no such bottleneck or that there has been strong differential selection on some locus that is tightly linked to this gene.

Such properties as LD decay are expected to be strongly influenced by the mating pattern of a population. For instance, soybean (Zhu *et al.* 2003) and wheat (Liu *et al.* 2010), two inbreeding species, were found to exhibit little LD decay within respective 50 kb and 3 cM regions, whereas outcrossing species like maize (Remington *et al.* 2001; Flint-Garcia *et al.* 2003) and *D. melanogaster* (Long *et al.* 1998) show exceptional decay within less than 1.5 kb. No significant decline in LD was found within the 2.3-kb and 4.6-kb regions analyzed for the two *rht1* and three *sd1* homologs, respectively, in agreement with the few tef haplotypes observed, the low frequency of heterozygotes, and the fact that tef is a preferential inbreeder for which even controlled crosses have been rarely accomplished (Berhe *et al.* 1989; Tefera *et al.* 2003).

Genome-wide LD studies in tef are needed to determine the average distances in which LD declines in this species. With the completion of the first detailed linkage map of tef (Yu *et al.* 2006), it will be possible to conduct association analysis studies in this species, once an assessment of average LD characteristics is made (Nordborg 2000).

### Gene and species divergence dates

The grass phylogeny predicted for the *rht1* data set is very similar to previous large-scale analyses that contained many more species (Gpwg 2001, Vicentini *et al.* 2008, Christin *et al.* 2008, Edwards *et al.* 2010), although the nodes at the base of the core grass clade (represented here by pooid, panicoid, ehrhartoid, and chloridoid grasses) lack support, as has also been seen in other single gene analyses (Devos *et al.* 2005). The estimated divergence times for species in the panicoid and chloridoid clades (including tef) using the *rht1* data set agree well with published figures (Devos *et al.* 2005, Vicentini *et al.* 2008), suggesting that the divergence dates between *tef-rht1-1* and *tef-rht1-2* are robust. However, the divergences dates of the pooid species (*Triticum*, *Hordeum*, and *Brachypodium*) in the *rht1* data set were consistently older than published studies (*Triticum*–*Brachypodium* 40 mya, *Triticum*–*Hordeum* 18 mya in the *rht1* analysis as compared with approximately 25 mya and 7 mya, respectively, in Vicentini *et al.* 2008 and Christin *et al.* 2008), although they were within the range of the confidence intervals. This may be because the calibration date of 55 million years that was used in this study is appropriate for the bamboos in addition to the subfamilies examined, but, unfortunately, no bamboo sequences were available for this study. The absence of these sequences may have affected the length of the tree, and it will be interesting to identify and incorporate *rht1* and *sd1* sequences from a bamboo species to examine effects on divergence dating.

The phylogeny predicted for the *sd1* data set suggests both duplication and subsequent loss of gene copies, resulting in a complex phylogenetic pattern. Several subclades in the analyses were consistently strongly supported, including a controversial placement of the three tef copies sister to rice rather than its more usual position of sister to the panicoid grasses. The three tef copies share unusual stretches of divergent sequences, both at the nucleotide and protein levels, but they do not contain stop codons, making it likely that these genes are functional. Differences in relationships between these subclades are sensitive to whether the data set is partitioned into codon positions, indicating that identifying the correct evolutionary model is crucial in understanding the evolution of this gene family. Further work is required to understand the phylogenetic relationships of the entire group of GA20 oxidases, in order that unequivocal orthology assessments can be made.

The divergence dates found for the tef *rht1* data set suggest that the allopolyploidization event that led to the two tef *rht1* copies dates to well before domestication of *E. tef* from *E. pilosa*. The human selection associated with tef domestication and improvement is likely to have contributed dramatically to narrowing the haplotype diversity in this crop and may also have selected for particular copy numbers of these gene families. Further analysis of these genes in wild *Eragrostis* species may help resolve these issues. Perhaps more important, analysis of the association between plant height and these genes in wild and domesticated *Eragrostis* accessions may provide access to gene copies useful for the generation of semidwarf tef varieties with improved lodging tolerance and the potential for a positive response to an increased input regimen.

## Supplementary Material

Supporting Information
