# Supplementary material for: Haplotype Analysis and Linkage Disequilibrium at Five Loci in Eragrostis tef
Source: G3 (Bethesda). 2012 Mar 1;2(3):407–19. doi: 10.1534/g3.111.001511 (PMC3291510; doi:10.1534/g3.111.001511)
Supplement: Supporting Information [file supp_2_3_407__index.html]

Supporting Information 

# Haplotype Analysis and Linkage Disequilibrium at Five Loci in *Eragrostis tef*

## Supporting Information for Smith *et al*, 2012

**Files in this Data Supplement:**

- Supporting Information - Figures S1 and S2 and Table S1 (PDF, 430 KB)
- Figure S1 - *rht1* maximum likelihood tree(PDF, 226 KB)
- Figure S2 - Maximum likelihood tree for *sd1* homologues (PDF, 209 KB)
- Table S1 - List of accessions used to construct the phylogenetic trees (PDF, 78 KB)
