# Supplementary material for: Haplotype Analysis and Linkage Disequilibrium at Five Loci in Eragrostis tef
Source: G3 (Bethesda). 2012 Mar 1;2(3):407–19. doi: 10.1534/g3.111.001511 (PMC3291510; doi:10.1534/g3.111.001511)
Supplement: Supporting Information [file supp_2.3.407_001511SI.pdf]

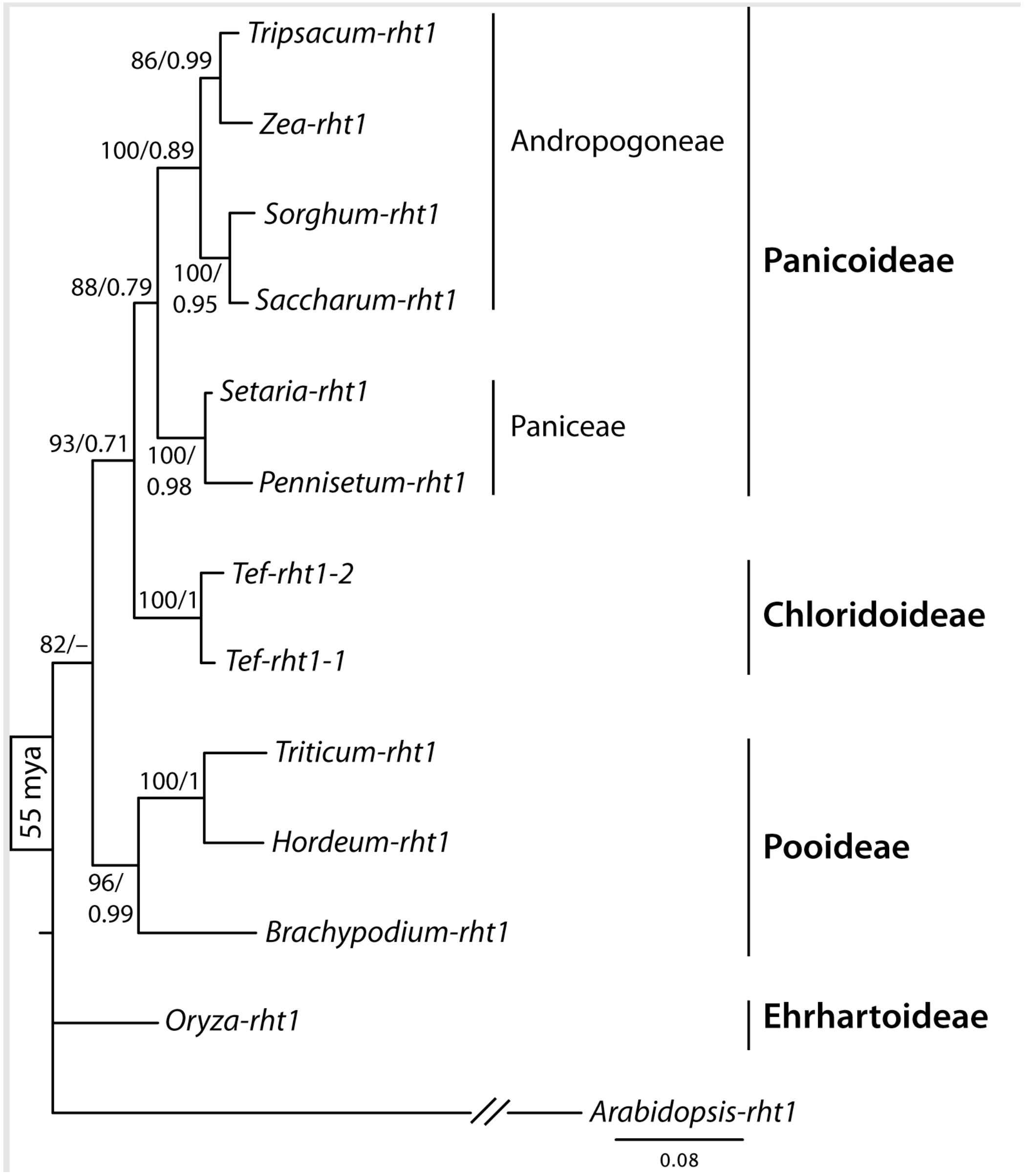

Figure S1 *rht1* maximum likelihood tree. Numbers on branches represent support values for clades (ML bootstrap support/Bayesian posterior probabilities). Box containing "55 mya" text indicates fossil calibration point for molecular dating. Taxon names indicate species. Vertical lines on right hand side indicate tribes (Andropogoneae and Paniceae) and subfamilies (Panicoideae, Chloridoideae, Pooideae, and Ehrhartoideae).

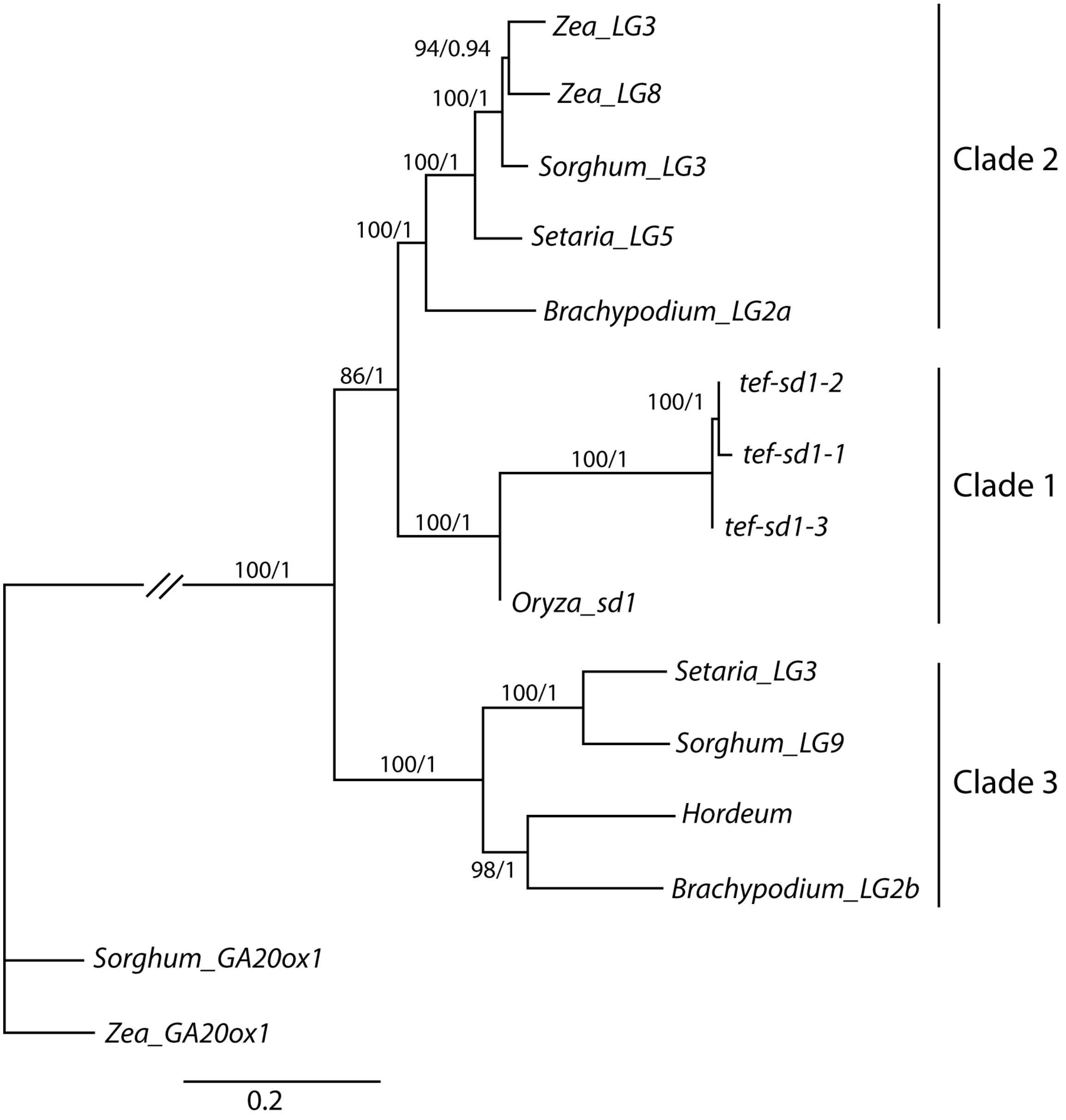

Figure S2 Maximum likelihood tree for *sd1* homologues. Numbers on branches represent support values for clades (ML bootstrap support/Bayesian posterior probabilities). Taxon names indicate species.

Table S1 List of accessions used to construct the phylogenetic trees

Tree label	Species	Accession Number
Rht1		
Tripsacum-rht1	*Tripsacum dactyloides*	AF377646 [a]
Zea-rht1	*Zea mays*	AJ242530 [a]
Sorghum-rht1	*Sorghum bicolor*	XM_002466549 [a]
Saccharum-rht1	*Saccharum officinarum*	DQ062091 [a]
Setaria-rht1	*Setaria italica*	Si039400 [b]
Pennisetum-rht1	*Pennisetum glaucum*	FJ011686 [a]
Tef-2-rht1	*Eragrostis tef*	JN793956 [a]
Tef-1-rht1	*Eragrostis tef*	JN793956 [a]
Triticum-rht1	*Triticum aestivum*	AJ242531 [a]
Hordeum-rht1	*Hordeum vulgare*	AF460219 [a]
Brachypodium-rht1	*Brachypodium distachyon*	Bradi1g11090 [b]
Oryza-rht1	*Oryza sativa*	AB262980 [a]
Arabidopsis-rht1	*Arabidopsis thaliana*	NM121755 [a]
Sd1		
Zea-LG3	*Zea mays*	Zm_GRMZM2G368411 [b]
Zea-LG8	*Zea mays*	Zm_GRMZM2G049418 [b]
Sorghum-LG3	*Sorghum bicolor*	XM_002456706 [a]
Setaria-LG5	*Setaria italica*	Si001573 [b]
Brachypodium-LG2a	*Brachypodium distachyon*	Bradi2g57030 [b]
Tef-sd1-2	*Eragrostis tef*	JN799335 [a]
Tef-sd1-1	*Eragrostis tef*	JN799304 [a]
Tef-sd1-3	*Eragrostis tef*	JN799366 [a]
Oryza-sd1	*Oryza sativa*	NM_001051549 [a]
Setaria-LG3	*Setaria italica*	Si025143 [b]
Sorghum-LG9	*Sorghum bicolor*	XM_002441072 [a]
Hordeum	*Hordeum vulgare*	AK373555 [a]
Brachypodium-LG2b	*Brachypodium distachyon*	Bradi2g24980 [b]
Sorghum-GA20ox1	*Sorghum bicolor*	XM_002463438 [a]
Zea-GA20ox1	*Zea mays*	EU969358 [a]

[a] Genbank accession numbers.
[b] Phytozome annotation numbers.
