# Supplementary material for: Haplotype Analysis and Linkage Disequilibrium at Five Loci in Eragrostis tef
Source: G3 (Bethesda). 2012 Mar 1;2(3):407–19. doi: 10.1534/g3.111.001511 (PMC3291510; doi:10.1534/g3.111.001511)
Supplement: Supporting Information [file supp_2.3.407_FigureS1.pdf]

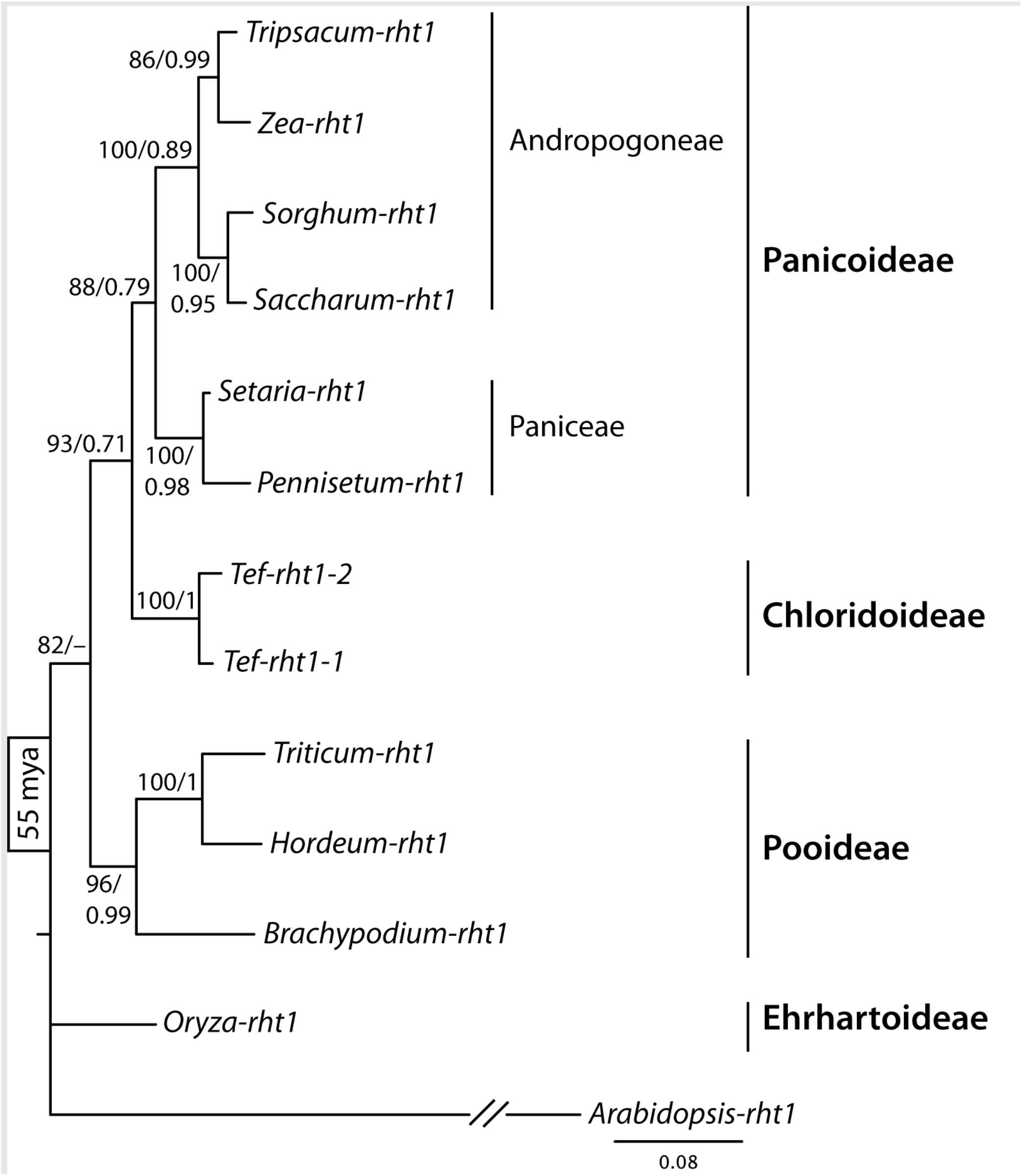

Figure S1 *rht1* maximum likelihood tree. Numbers on branches represent support values for clades (ML bootstrap support/Bayesian posterior probabilities). Box containing “55 mya” text indicates fossil calibration point for molecular dating. Taxon names indicate species. Vertical lines on right hand side indicate tribes (Andropogoneae and Paniceae) and subfamilies (Panicoideae, Chloridoideae, Pooideae, and Ehrhartoideae).
