# Supplementary material for: Haplotype Analysis and Linkage Disequilibrium at Five Loci in Eragrostis tef
Source: G3 (Bethesda). 2012 Mar 1;2(3):407–19. doi: 10.1534/g3.111.001511 (PMC3291510; doi:10.1534/g3.111.001511)
Supplement: Supporting Information [file supp_2.3.407_FigureS2.pdf]

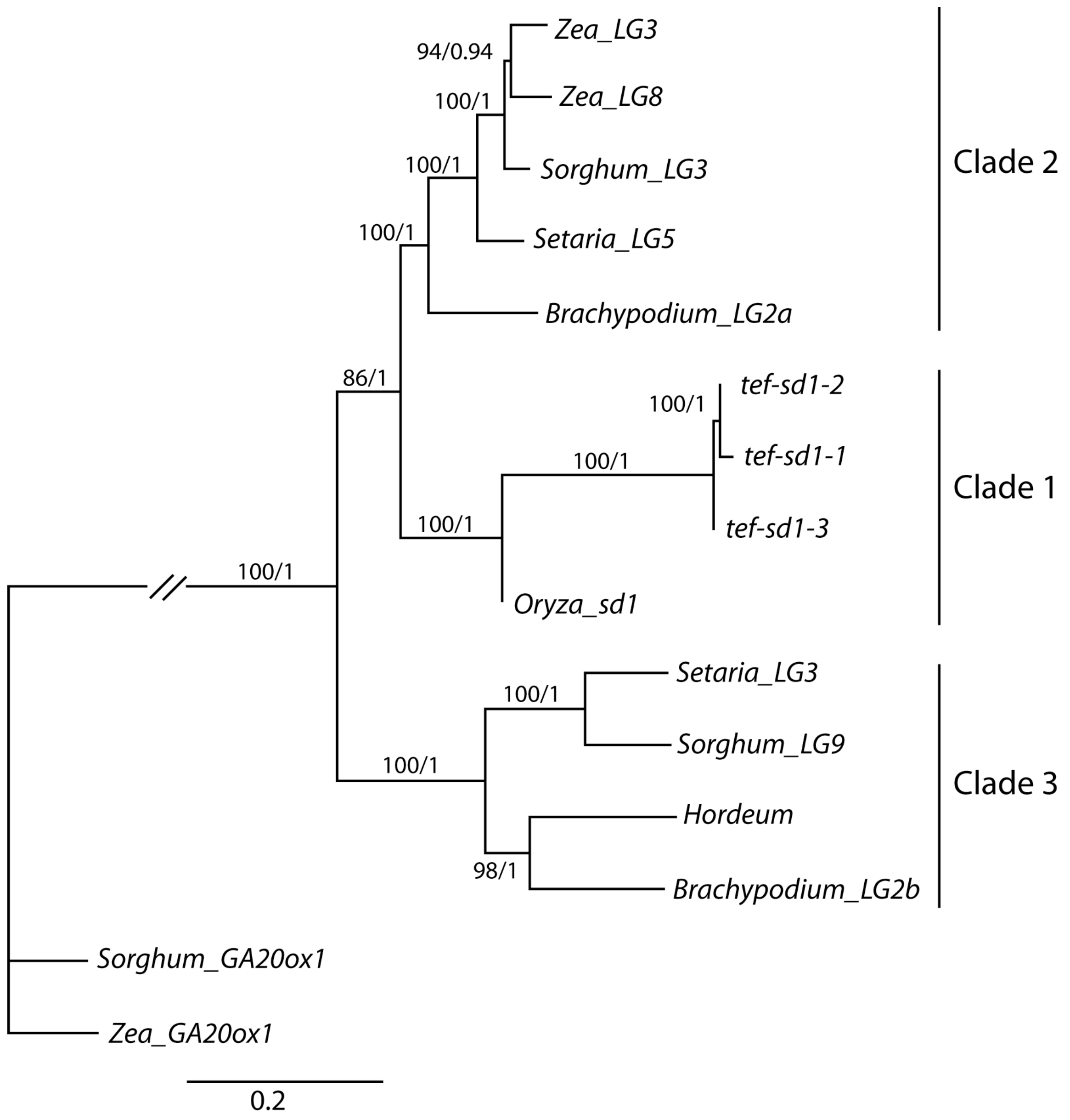

Figure S2 Maximum likelihood tree for *sd1* homologues. Numbers on branches represent support values for clades (ML bootstrap support/Bayesian posterior probabilities). Taxon names indicate species.
