# Supplementary material for: Haplotype Analysis and Linkage Disequilibrium at Five Loci in Eragrostis tef
Source: G3 (Bethesda). 2012 Mar 1;2(3):407–19. doi: 10.1534/g3.111.001511 (PMC3291510; doi:10.1534/g3.111.001511)
Supplement: Supporting Information [file supp_2.3.407_TableS1.pdf]

Table S1 List of accessions used to construct the phylogenetic trees

Tree label	Species	Accession Number
Rht1		
Tripsacum-rht1	*Tripsacum dactyloides*	AF377646 [a]
Zea-rht1	*Zea mays*	AJ242530 [a]
Sorghum-rht1	*Sorghum bicolor*	XM_002466549 [a]
Saccharum-rht1	*Saccharum officinarum*	DQ062091 [a]
Setaria-rht1	*Setaria italica*	Si039400 [b]
Pennisetum-rht1	*Pennisetum glaucum*	FJ011686 [a]
Tef-2-rht1	*Eragrostis tef*	JN793956 [a]
Tef-1-rht1	*Eragrostis tef*	JN793956 [a]
Triticum-rht1	*Triticum aestivum*	AJ242531 [a]
Hordeum-rht1	*Hordeum vulgare*	AF460219 [a]
Brachypodium-rht1	*Brachypodium distachyon*	Bradi1g11090 [b]
Oryza-rht1	*Oryza sativa*	AB262980 [a]
Arabidopsis-rht1	*Arabidopsis thaliana*	NM121755 [a]
Sd1		
Zea-LG3	*Zea mays*	Zm_GRMZM2G368411 [b]
Zea-LG8	*Zea mays*	Zm_GRMZM2G049418 [b]
Sorghum-LG3	*Sorghum bicolor*	XM_002456706 [a]
Setaria-LG5	*Setaria italica*	Si001573 [b]
Brachypodium-LG2a	*Brachypodium distachyon*	Bradi2g57030 [b]
Tef-sd1-2	*Eragrostis tef*	JN799335 [a]
Tef-sd1-1	*Eragrostis tef*	JN799304 [a]
Tef-sd1-3	*Eragrostis tef*	JN799366 [a]
Oryza-sd1	*Oryza sativa*	NM_001051549 [a]
Setaria-LG3	*Setaria italica*	Si025143 [b]
Sorghum-LG9	*Sorghum bicolor*	XM_002441072 [a]
Hordeum	*Hordeum vulgare*	AK373555 [a]
Brachypodium-LG2b	*Brachypodium distachyon*	Bradi2g24980 [b]
Sorghum-GA20ox1	*Sorghum bicolor*	XM_002463438 [a]
Zea-GA20ox1	*Zea mays*	EU969358 [a]

[a] Genbank accession numbers.
[b] Phytozome annotation numbers.
